# Priorities in healthcare provision in Parkinson's disease from the perspective of Parkinson Nurses: A focus group study

**DOI:** 10.1016/j.ijnsa.2024.100213

**Published:** 2024-06-12

**Authors:** Marlena van Munster, Katarzyna Czabanowska, Timo Clemens, Estera Wieczorek, David Pedrosa, Tiago A. Mestre, Johanne Stümpel

**Affiliations:** a Department of Neurology, Philipps University Marburg, 35043 Marburg, Germany; b Institute of General Medicine, Charité - Universitätsmedizin Berlin, Berlin, Germany; c Department of International Health, CAPHRI Care and Public Health Research Institute, Maastricht University, 6229 ER Maastricht, Netherlands; d Department of Health Policy Management, Institute of Public Health, Faculty of Health Sciences, Jagiellonian University, 31-066 Krakow, Poland; e Department of Health Economics and Social Security, Institute of Public Health, Faculty of Health Sciences, Jagiellonian University Collegium Medicum, Krakow, Poland; f Department of Health Services Research, Faculty of Health, Medicine and Life Sciences, CAPHRI, Maastricht University, Maastricht, Netherlands; g Parkinson Disease and Movement Disorders Centre, Division of Neurology, Department of Medicine, The Ottawa Hospital Research Institute, University of Ottawa Brain and Mind Research Institute, Ottawa, ON K1Y 4E9, Canada; h Center for Life Ethics, University of Bonn, 53113 Bonn, Germany

**Keywords:** Parkinson's disease, Nursing, Parkinson Nurse, Competence, Healthcare utilization

## Abstract

**Background:**

Through their expertise and diverse skills, Parkinson Nurses are key care providers for people with Parkinson's disease. They are seen as an important profession for person-centered and multidisciplinary care, considered priorities in Parkinson's care delivery. Currently, however, little is known about the priorities that this profession itself defines for the care of Parkinson's patients and how they perceive their own role in the care process.

**Objective:**

To explore the perspective of Parkinson Nurses on care priorities in people with Parkinson's disease.

**Design:**

Qualitative study.

**Setting(s):**

The iCare-PD study served as the object of study by establishing an interdisciplinary, person-centered and nurse-led care model in several European countries and Canada. The nurses who participated in this model were part of the study.

**Participants:**

Six Parkinson Nurses participated in the study.

**Methods:**

We conducted a thematic focus group, adopting the paradigm of pragmatism to draft an interview guide. The focus group was based on the inspiration card method and followed recommendations for co-creation processes.

**Results:**

Parkinson Nurses define care priorities for Parkinson's in areas of education, multi-professionalism, and need-orientation. They see themselves as mediators and coordinators of care delivery processes.

**Conclusions:**

In line with international recommendations, Parkinson Nurses prioritize key aspects of multidisciplinary and person-centered care. At the same time, however, the nurses also name care priorities that go beyond the international recommendations. It is therefore crucial to integrate the perspective of this important profession into recommendations for the delivery of healthcare for people with Parkinson's.

Tweetable abstract How do *specialized nurses define priorities for person-centered Parkinson's care? Answers are sought in this qualitative study by @MarlenaMunster.*

**What is already known**

• International recommendations and priorities in Parkinson's care delivery often focus on person-centered and multidisciplinary care.
• Parkinson Nurses are vital in providing care for individuals with Parkinson's by person-centered and multidisciplinary approaches.
• Despite the recognized importance of Parkinson Nurses, there is a gap in understanding their specific care priorities and perceptions of their role in the person-centered care process.

**What this paper adds**

• This paper sheds light on the care priorities identified by Parkinson Nurses, emphasizing areas such as education, multi-professionalism, and need-orientation.
• Parkinson Nurses view themselves as central mediators and coordinators in delivering care for individuals with Parkinson's disease, shaping the landscape of person-centered, multidisciplinary care.
• The findings highlight the importance of integrating the perspectives of Parkinson Nurses into healthcare recommendations to ensure comprehensive and effective care delivery for Parkinson's patients.

## 1 Background

Parkinson's disease is a neurodegenerative disorder characterized by complex multimorbid motor and non-motor signs and symptoms, typically affecting individuals over the age of 60. In Europe, approximately 1.2 million people are currently living with Parkinson's disease, and its incidence is rising among the elderly population (Gustavsson et al. 2011). Globally, the number of individuals affected by Parkinson's disease is projected to double by 2030 (Dorsey et al. 2007).

As the prevalence of patients with co-morbidities rises within healthcare systems, including those with Parkinson's disease, there is a growing need for tailored care structures to address their unique requirements (Palladino et al. 2016). People with Parkinson's disease are considered particularly vulnerable to inefficient care structures (Zaman, Ghahari, and McColl 2021). International guidelines recommend integrated, person-centered, and multidisciplinary approaches to Parkinson's care, and recognize the vital role of specialized nurses, known as Parkinson Nurses, in providing comprehensive and individualized support (Lidstone, Bayley, and Lang 2020; Radder et al. 2019; Rajan et al. 2020).

Parkinson Nurses take on a variety of tasks that contribute to a more person-centered approach (van Munster et al. 2022). Furthermore, they are seen as a key profession in the establishment of multidisciplinary care delivery (Radder et al. 2019). Despite their significance, there remains a limited understanding of the specific care priorities identified by Parkinson Nurses and their perception of their role in the broader care delivery landscape. This research seeks to bridge this gap by examining the care priorities designated by Parkinson Nurses and gaining insights into their perceptions of their profession's role in delivering healthcare to individuals with Parkinson's disease within a multinational integrated care model.

## 2 Methods

### 2.1 Study design and theoretical background

This qualitative research is grounded in the paradigm of pragmatism, which sees human experience as central to building knowledge and understanding the world (Allemang et al., 2022). This research paradigm is well suited to exploring complex issues and subjective perspectives in health research (Allemang, Sitter, and Dimitropoulos 2022). A qualitative research approach was chosen to capture this experience. Qualitative research methods are considered appropriate in health research to investigate complex issues and subjective perspectives (Powell and Single 1996). In this context, the focus group technique was chosen to explore nurses' perceptions and attitudes effectively. In particular, focus groups are suitable for exploring perceptions and attitudes (Powell and Single 1996).

To capture the perspective of nurses, it was relevant to form a focus group with professionals who have a broad perspective on care delivery for people with Parkinson's disease. A purposive sampling approach was employed to ensure a comprehensive understanding of Parkinson's care delivery, integrating six nurses from Canada and various European countries (Germany, Portugal, Italy, Czech Republic, Ireland) involved in a novel care delivery model (Fabbri et al. 2020). Data collection occurred in Portugal in 2022, with nurses participating either in person or online. These nurses assumed the role of care coordinators within a person-centered, multidisciplinary care concept, making them well-suited for providing insights (Mestre et al. 2021; van Munster et al. 2021).

**Fig. 1:**
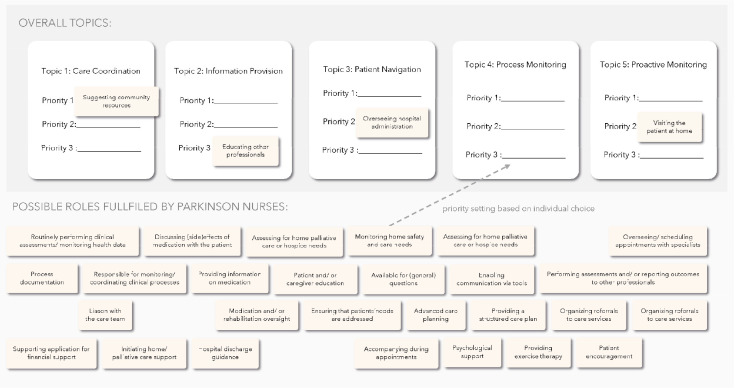
Illustration of the inspiration card method for stimulating group discussion. The overall topics correspond to the theory of person-centered Parkinson's care according to (van Halteren et al. 2020) and the roles fulfilled by a Parkinson Nurse are adapted from the review by (van Munster et al. 2022).

The focus group discussions were facilitated using the inspiration card method, encouraging active participation and stimulating discussions around key priorities in Parkinson's care delivery (Halskov and Dalsgård 2006). A schematic representation of this process is shown in Fig. 1.

The cards presented various tasks typically undertaken by nurses in multidisciplinary and person-centered Parkinson's care models, adapted from van Munster et al. (van Munster et al. 2022). The moderators identified five overarching themes, guided by the elements of person-centered Parkinson's care outlined by van Halteren et al. (van Halteren et al. 2020) . After reviewing the cards, nurses were prompted to select and justify the three most important priorities for each top theme based on their perspectives. Facilitators (M.vM., Public Health, M.Sc., a female researcher based in Germany; and J.S., M.Sc., a female researcher and health economist based in Germany) guided the conversation by asking specific questions to deepen explanations, fostering a rich and insightful dialogue among participants.

### 2.2 Data collection

Completed cards were collected from participants and archived as part of the study results. Pseudo-anonymized analysis and stringent data protection measures were implemented, including removing any identifying information, such as names or specific locations. Additionally, an audio recording of the group discussion was transcribed verbatim to capture all dialogue accurately.

### 2.3 Data analysis

Following transcription, audio recordings underwent thorough accuracy verification. Focus group data analysis was conducted by two proficient team members (M.vM. and J.S.) utilizing Braun and Clarke's thematic analysis method (Clarke, Braun, and Hayfield 2015). To ensure result credibility, initial coding was independently performed by the research team. Both researchers possess extensive expertise in qualitative research, specifically in Parkinson's care and Parkinson's Nursing. Analysis involved a multi-stage process of category development and text segment coding. Initially, data were deductively coded based on research questions, forming categories reflective of factors shaping Parkinson Nurses' perspectives. The initial data analysis was informed by two research questions: (1) What care priorities do Parkinson Nurses define in terms of person-centred Parkinson's care? (2) How do Parkinson Nurses perceive their role in person-centred Parkinson's care? Inductive coding further refined these categories. Subsequent data verification and credibility checks were conducted collaboratively, with discrepancies resolved through discussion. MAXQDA (version 2020) was used to facilitate qualitative coding.

## 3 Results

In total, the group is very small, which is why the nurses' wish to remain anonymous was respected. No personal data was published. Six Parkinson Nurses participated in the workshop. The workshop lasted 1 h and 30 min.

### 3.1 Overall themes

**Fig. 2:**
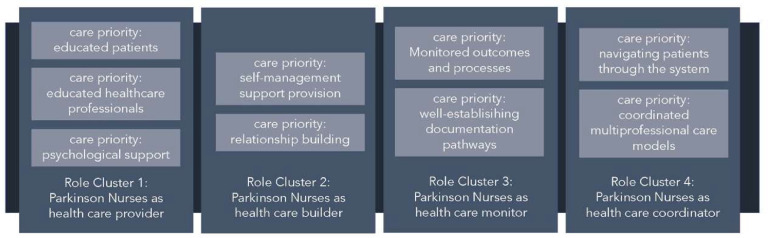
Overall themes of the coding process.

The coding process resulted in four overarching clusters in which the Parkinson Nurses fulfilled their role in the care process: delivering, setting up, measuring, and coordinating. During the coding process, care priorities from the perspective of the Parkinson Nurses could be assigned to these four role clusters. A detailed description of the coding results can be found in the supplementary material (Table A1) (Fig. 2).

### 3.2 The role of a health care provider

Nurses define the provision of education, both to those affected, in the sense of patients and relatives, and to other professionals as an important priority in Parkinson's Nursing practice. Patient education is seen as key to initiating discussion about support options:

*“[…] we try to do, like before they go to their homes, we try to see and to speak and we have a meeting trying to see what we can, they do like to buy or something, and to prevent and to proactively trying to prevent something.”* (PN1)

Educating professionals, on the other hand, aims more at changing professional actions. This is seen primarily as a way of enabling patients to make better use of the necessary care services:

*“[…] one of the things we try to do is always educate other healthcare providers, either in the hospital or the community setting. Because at the end of the day, they will be the ones taking care of these patients.”* (PN3)

Nurses perceive psychological support as another important aspect of provision. In this context, they reflect the special closeness to the patient as an advantage that allows them to empower patients:

*“I guess that you give indirectly psychological support to the patient […] which is something that sometimes they really need, because otherwise they don't understand what can happen next. They cannot have a proactive approach to anything, […]”* (PN5)

### 3.3 The role of a health care builder

Nurses perceive themselves as holding an active role in the process of building a relationship with affected persons and forming self-management skills in the affected persons.

*“I also put it, patient encouragement. […] try to make them search and think about other stuff, and giving the tools so they can actually process it themselves.”* (PN1)

In doing so, they also describe that these processes are resource intensive:

*“But really practically speaking, you actually need time. To, as I told you before, to create this relationship with the patient, to create your background, to specialize, to understand, to know the patients, you need time.”* (PN6)

### 3.4 The role of a health monitor

Nurses define priorities of their actions on both the content (i.e., what they monitor) and process (i.e., how they monitor) levels. In terms of content, medication, and the needs of those affected at home are the main priorities. Monitoring these aspects is associated with better utilization of care:

*“[…] what we would do is they would go to the wards where they're being treated and make sure that their medications are correct and that they're getting the proper support, […]”* (PN4)

*“I put the visiting the patient at home. […] they feel more comfortable and sometimes they speak some things to me that they don't speak with the doctor at the hospitals, because they're so stressed out because they have to go to the hospital. […] it's easier sometimes for them to speak out and sometimes even getting the information.”* (PN3)

With regard to the "how", nurses perceive their role primarily in structured recording, which is considered a good basis of information for care planning:

*“[…] you need to be a bit structured for me. […] actually, having a routine questionnaire session can be useful also because you can appreciate the differences and you can discuss with the patient about that of course. […] So actually, this assessment or health data can be really important.”* (PN6)

### 3.5 The role of a health care coordinator

Nurses perceive the coordination of care pathways and thus the coordination of patient's health care utilization as one of their most central roles. In this context, coordination refers to pathways both within and outside the organizations in which the nurses work:

*“I chose hospital discharge guidance. The main reason why I chose that is because, as I said, the Parkinson nurse specialist here, what they do is that they liaise between the acute hospitals and the community services.”* (PN2)

*“[…] what we try to do is we try to communicate to their GPs so that the person that they see out in the community and use the GP as a resource in that to identify community resources that are available to them.”* (PN1)

Nurses also see themselves as coordinators of various professions within the care process:

*“I choose providing a structured care plan, liaison with the care team, and ensuring that patients' needs are addressed. Of course, the care team for me is absolutely important because we know that the patients’ needs are so many and we have to, if we want to ensure that their needs are addressed, we need to have a super strong network.”* (PN4)

### 3.6 Wishes for improvement

However, the nurses also perceive limitations in fulfilling their prioritized roles and defined improvements that, from their perspective, would help them to better fulfil their roles according to their priorities. For participants from four countries the structure of care in their own country was a limitation, in which their role is not foreseen:

*“[…] first I put that there should be a nurse maybe as a responsible for monitoring and coordinating all the clinical processes. […] we don't have it and we have to start from the basis, which means to start with a person, […] as responsible of this process […].”* (PN2)

In addition, all participants expressed the desire for a common set of competence standards to make their profession clearly tangible:

*“[…] the hospital needs to, or the government needs to acknowledge that it (“the role Parkinson Nurse”) is important and that even the healthcare members need to acknowledge what this role does and how it fits within the whole picture of the healthcare system.”* (PN3)

*“Like a training structure as well. It would be nice because that kind of shows you like, "This is what you need really. These are the skills that are important for a Parkinson Nurse. Whether it be inpatient based or outpatient based, but these are the kind of core skills you need to have."* (PN6)

## 4 Discussion

The central tenets of future Parkinson's care are multi-professionalism and need-orientation (Achey et al. 2014; Rajan et al. 2020). Research indicates that adopting person-centered approaches can enhance patients' utilization of healthcare services, albeit necessitating a coordinating entity (Bhidayasiri et al. 2020). Notably, nurses perceive themselves as pivotal mediators between patients and the wider care team, an insight crucial for sustaining Parkinson's care (Tenison, James, Ebenezer, and Henderson 2022). The perceptions of these roles align with contemporary recommendations for the organization of multi-professional care strategies in Parkinson's disease (Radder et al. 2019).

However, the expectations of a nurses' role are evolving beyond conventional professional paradigms, influenced by project-based training and practical experiences. In Germany, while training programs for Parkinson Nurses exist, the profession itself is not formally recognized, leading to integration within traditional care frameworks (Mai 2018). Consequently, roles such as coordinating care pathways within institutions or providing home-based care, highlighted in this study, remain underutilized (Mai 2018).

Despite international recognition of the importance of the Parkinson Nurse role (van Munster et al. 2022), its establishment remains limited to selected countries globally. Theoretical frameworks emphasize the significance of identifying one's professional actions and delineating a clear competency profile as foundational elements of a profession (Williams 1998). Findings from this study suggest that expanding competency profiles could enhance awareness and professional recognition.

From a practical standpoint, this study implies that nurses, when equipped with expanded competencies, can redefine their role and contribute to the advancement of forward-looking care models. However, it also underscores the necessity for tailored training programs and practical opportunities to enable nurses to effectively apply these competencies.

## 5 Limitations

While the qualitative focus group with nurses from diverse backgrounds working on the same integrated care delivery project offers valuable insights, several potential limitations should be acknowledged. Firstly, the small sample size of six participants may limit the generalizability of findings to a broader population of Parkinson Nurses. Additionally, participants from different countries represent varying healthcare systems and cultural contexts, which may influence their perspectives and priorities, potentially leading to findings that are specific to this group. Furthermore, the qualitative nature of the study may introduce subjectivity and researcher bias into data analysis, though rigorous methodological approaches can mitigate this. Finally, as with any qualitative research, the depth of understanding may be constrained by the time limitations of a single focus group session. These limitations should be considered when interpreting and applying the study findings.

## 6 Conclusions

In conclusion, this qualitative study provides critical insights into the perspectives and priorities of Parkinson Nurses for individuals with Parkinson's disease. These findings hold significant implications for the delivery of healthcare services and resources. The nurses' emphasis on education, multi-professional collaboration, need-orientation, and their roles as mediators and coordinators underscores the importance of a comprehensive and patient-centered approach in Parkinson's care. By recognizing and integrating these priorities into healthcare systems, organizations can harness the potential to further develop healthcare resources.

Although limitations such as a small sample size exist, these findings flag to healthcare providers and policy makers the need to re-evaluate and adapt their approaches to Parkinson's care and call for a recognition of professional role development as important part of healthcare development.

## Ethical considerations

According to the guidelines of the German Research Foundation, a separate ethical approval' was not required for this sub-study of the iCare-PD project. The iCare-PD project was approved by the responsible ethics committee of the University of Marburg (reference: 164/19).

## Funding sources

This research was funded as part of the research project “iCARE-PD”. This is an EU Joint Programme Neurodegenerative Disease Research (JPND) project (Reference Number: HESOCARE-329-073). The project is supported through the following funding organizations under the aegis of JPND—*www.jpnd.eu* (accessed on 1st July 2022): Canada—Canadian Institutes of Health Research; Czech Republik—Ministry of Education, Youth and Sport of the Czech Republic; France—Agence National de la Recherche; Germany—Bundesministerium für Bildung und Forschung; Spain—National Institute of Health Carlos III; United Kingdom—Medical Research Council. M.v.M. and J.S. are funded by the research project “iCARE-PD”.

## CRediT authorship contribution statement

**Marlena van Munster:** Writing – review & editing, Writing – original draft, Visualization, Validation, Methodology, Investigation, Formal analysis, Conceptualization. **Katarzyna Czabanowska:** Writing – review & editing, Supervision. **Timo Clemens:** Writing – review & editing, Supervision. **Estera Wieczorek:** Writing – review & editing. **David Pedrosa:** Writing – review & editing, Supervision, Resources, Funding acquisition. **Tiago A. Mestre:** Writing – review & editing, Supervision, Funding acquisition. **Johanne Stümpel:** Writing – review & editing, Writing – original draft, Methodology, Investigation, Formal analysis, Conceptualization.

## Declaration of competing interest

The authors declare that they have no known competing financial interests or personal relationships that could have appeared to influence the work reported in this paper.

## Appendix Supplementary materials

Image, application 1

## References

[bib0001] Achey M., Aldred J.L., Aljehani N., Bloem B.R., Biglan K.M., Chan P., Cubo E., Dorsey E.R., Goetz C.G., Guttman M., Hassan A., Khandhar S.M., Mari Z., Spindler M., Tanner C.M., van den Haak P., Walker R., Wilkinson J.R. (2014). The past, present, and future of telemedicine for Parkinson's disease. Mov. Disord..

[bib22] Allemang B., Sitter K., Dimitropoulos G. (2022). Pragmatism as a paradigm for patient-oriented research. Health Expect..

[bib0002] Allemang B., Sitter K., Dimitropoulos G. (2022). Pragmatism as a paradigm for patient-oriented research. Health Expect..

[bib0003] Bhidayasiri R., Panyakaew P., Trenkwalder C., Jeon B., Hattori N., Jagota P., Wu Y.R., Moro E., Lim S.Y., Shang H., Rosales R., Lee J.Y., Thit W.M., Tan E.K., Lim T.T., Tran N.T., Binh N.T., Phoumindr A., Boonmongkol T., Phokaewvarangkul O., Thongchuam Y., Vorachit S., Plengsri R., Chokpatcharavate M., Fernandez H.H. (2020). Delivering patient-centered care in Parkinson's disease: Challenges and consensus from an international panel. Parkinsonism Relat. Disord..

[bib0004] Clarke Victoria, Braun Virginia, Hayfield Nikki (2015). Thematic analysis. Qualitative psychology: A practical guide to research methods.

[bib0005] Dorsey E.R., Constantinescu R., Thompson J.P., Biglan K.M., Holloway R.G., Kieburtz K., Marshall F.J., Ravina B.M., Schifitto G., Siderowf A., Tanner C.M. (2007).

[bib0006] Fabbri M., Caldas A.C., Ramos J.B., Sanchez-Ferro Á, Antonini A., Růžička E., Lynch T., Rascol O., Grimes D., Eggers C., Mestre T.A., Ferreira J.J. (2020). Moving towards home-based community-centred integrated care in Parkinson's disease. Parkinsonism Relat. Disord..

[bib0007] Gustavsson A., Svensson M., Jacobi F., Allgulander C., Alonso J., Beghi E., Dodel R., Ekman M., Faravelli C., Fratiglioni L., Gannon B., Jones D.H., Jennum P., Jordanova A., Jonsson L., Karampampa K., Knapp M., Kobelt G., Kurth T., Lieb R., Linde M., Ljungcrantz C., Maercker A., Melin B., Moscarelli M., Musayev A., Norwood F., Preisig M., Pugliatti M., Rehm J., Salvador-Carulla L., Schlehofer B., Simon R., Steinhausen H.C., Stovner L.J., Vallat J.M., Van den Bergh P., van Os J., Vos P., Xu W., Wittchen H.U., Jonsson B., Olesen J. (2011). Cost of disorders of the brain in Europe 2010. Eur. Neuropsychopharmacol..

[bib0008] Halskov Kim, Dalsgård Peter (2006). Proceedings of the 6th conference on Designing Interactive systems.

[bib0009] Lidstone Sarah C., Bayley Mark, Lang Anthony E. (2020). The evidence for multidisciplinary care in Parkinson's disease. Expert. Rev. Neurother..

[bib0010] Mai Tobias. (2018). Stand und entwicklung der rolle als Parkinson Nurse in Deutschland–eine online-befragung. Pflege.

[bib0011] Mestre T.A., Kessler D., Cote D., Liddy C., Thavorn K., Taljaard M., Grimes D. (2021). Pilot Evaluation of a Pragmatic Network for Integrated Care and Self-Management in Parkinson's Disease. Mov. Disord..

[bib0012] Palladino Raffaele, Lee John Tayu, Ashworth Mark, Triassi Maria, Millett Christopher (2016). Associations between multimorbidity, healthcare utilisation and health status: evidence from 16 European countries. Age Ageing.

[bib0013] Powell Richard A., Single Helen M. (1996). Focus Groups'. Int. J. Qual. Health Care.

[bib0014] Radder Danique L.M., Vries Nienke M.de, Riksen Niels P., Diamond Sarah J., Gross Ditza, Gold Daniel R., Heesakkers John, Henderson Emily, Hommel Adrianus L.A.J., Lennaerts Herma H., Busch Jane, Dorsey Ray E., Andrejack John, Bloem Bastiaan R. (2019). Multidisciplinary care for people with Parkinson's disease: the new kids on the block!. Expert. Rev. Neurother..

[bib0015] Rajan R., Brennan L., Bloem B.R., Dahodwala N., Gardner J., Goldman J.G., Grimes D.A., Iansek R., Kovacs N., McGinley J., Parashos S.A., Piemonte M.E.P., Eggers C. (2020). Integrated care in Parkinson's Disease: a systematic review and meta-analysis. Mov. Disord..

[bib0016] Tenison Emma, James Alice, Ebenezer Louise, Henderson Emily J. (2022). A narrative review of specialist Parkinson&rsquo;s nurses: evolution, evidence and expectation. Geriatrics.

[bib0017] van Halteren Angelika D., Munneke Marten, Smit Eva, Thomas Sue, Bloem Bastiaan R., Darweesh Sirwan K.L. (2020). Personalized care management for persons with Parkinson's disease'. J. Parkinson's Disease.

[bib0018] van Munster M., Stümpel J., Thieken F., Ratajczak F., Rascol O., Fabbri M., Clemens T., Czabanowska K., Mestre T.A., Pedrosa D.J. (2022). The role of Parkinson Nurses for personalizing care in Parkinson's disease: a systematic review and meta-analysis. J. Parkinsons Dis.

[bib0019] van Munster Marlena, Stümpel Johanne, Thieken Franziska, Pedrosa David J., Antonini Angelo, Côté Diane, Fabbri Margherita, Ferreira Joaquim J., Růžička Evžen, Grimes David, Mestre Tiago A. (2021). Moving towards integrated and personalized care in Parkinson's disease: a framework proposal for training Parkinson Nurses. J. Personalized Med..

[bib0020] Williams Jerry L. (1998). What makes a profession a profession?. Prof. Saf..

[bib0021] Zaman M.S., Ghahari S., McColl M.A. (2021). Barriers to accessing healthcare services for people with Parkinson's disease: a scoping review. J. Parkinsons Dis.

