# Supplementary material for: Priorities in healthcare provision in Parkinson's disease from the perspective of Parkinson Nurses: A focus group study
Source: Int J Nurs Stud Adv. 2024 Jun 12;7:100213. doi: 10.1016/j.ijnsa.2024.100213 (PMC11332201; doi:10.1016/j.ijnsa.2024.100213)
Supplement: Supplementary file 1 [file mmc1.docx]

**Appendix**

Table A 1 - Detailed overview of the code tree.

| Role Cluster #1: Parkinson Nurses as health care providers… |
| --- |
| … education to those affected by Parkinson´s disease (n=2). |
| *“I chose the home safety and care needs monitoring. Because actually our Parkinson’s patient, they have […] a very high risk of falling, and if we can adapt and teach them and the family how to arrange their houses, it's something that we can proactively do and try to prevent falls […] and other stuff.” (PN2)*  *“I'm doing home visits also. So, I go there and sit with them and also their family, and see ‘what can we do’? What can we actually, like, try to adapt to be easier for them and also for their caregivers. Because sometimes that's just a little bit of a change and it's something that can make them comfortable. […] In here we try to do the same, like before they go to their homes, we try to see and to speak and we have a meeting trying to see what can we, they do, […] to proactively try to prevent something.” (PN1)* |
| … education to other healthcare professionals (n=2). |
| *“My priority is actually educating other professionals because […] if we are not connected as professionals, we will never change anything. So, for me, starting from our personal situation in country X, educating other professionals who […] normally don't actually care or specialize in Parkinson's, that would be the priority.” (PN1)*  *“[…] I think it's similar here in country X, since there's so many limited posts of Parkinson’s nurse specialists, one of the things we try to do is always educate other healthcare providers, either in the hospital or the community setting. Because at the end of the day, they will be the ones taking care of these patients. If we promote more information of how Parkinson's progresses, what medications and how to change medications, and how to address the problems that Parkinson's patient face, that's the most feasible way of addressing the lack of manpower to drive Parkinson's care in a country.” (PN3)* |
| … psychological support to those affected by Parkinson´s disease (n=5). |
| *“Yeah […] the […] documentation aspect, that is it. Then also booking them again for the next visit. So, the routine actually […] the planning as well. I think […] very broadly, like psychological support. That's, again, from my point of view, because I'm dealing a lot with new patients or met with patients with early onset, and they're becoming very stressed out. They don't know what to do, everything is new. So that's important as well.” (PN3)*  *“Lots of people that I met, they needed (it). […] In the first year that they find out, they ask me, they would like to talk, because they don't talk about that with relatives, family, and work. They don't want to say anything to anybody. But they still need to talk about that to somebody. So, we talk to them and then we offer them of course the psychological or psychiatric help.” (PN4)*  *“I guess that you give indirectly psychological support to the patient because it can be more relaxing and it can make them be more conscious of the illness, which is something that sometimes they really need, because otherwise they don't understand what can happen next. They cannot have a proactive approach to anything […] it's all related […] but the patients have to be involved in that process.” (PN5)*  *“I would agree with everything what was said, like understanding patient. And also, what I think is important is to give them hope as well, because at the beginning, […] it can be depressing and to give them hope, like to show them it's not the end. They can live happily.” (PN6)*  *“As I'd said so far, you have to be very empathetic and you have to be, I think, really patient with dealing with these Parkinson's patients because it's quite debilitating and some of them take a lot of time to communicate themselves, and you have to really listen.” (PN1)* |
| Role Cluster #2: Parkinson Nurses as health care builders… |
| …of self-management skills in those affected by Parkinson´s disease (n=3). |
| *“I guess that you give indirectly psychological support to the patient because it can be more relaxing and it can make them be more conscious of the illness, which is something that sometimes they really need, because otherwise they don't understand what can happen next. They cannot have a proactive approach to anything […] it's all related […] but the patients have to be involved in that process.” (PN2)*  *“I also put it, patient encouragement. Because besides giving information, we try to stimulate them for trying to see good websites […] try to make them search and think about other stuff, and giving the tools so they can actually process it themselves.” (PN1)*  *“I would agree with everything what was said, like understanding patient. And also, what I think is important is to give them hope as well, because at the beginning, […] it can be depressing and to give them hope, like to show them it's not the end. They can live happily.” (PN4)* |
| ...of relationships with those affected by Parkinson´s disease (n=2). |
| *“I think it's a mix of expertise and empathy and trust to be responsible. I think it's important to have the ability to separate one's self internally in order to remain clear in contact with a participant or the patient.” (PN3)*  *“But really practically speaking, you actually need time […] to create this relationship with the patient, to create your background, to specialize, to understand, to know the patients, you need time.” (PN6)* |
| Role Clusters #3: Parkinson Nurses as health care monitors… |
| …of patients’ medication (n=2). |
| *“Of the kind of main tasks for them is that when a patient gets admitted in the hospital, so what our PD nurse specialist, what they would do is they would go to the wards where they're being treated and make sure that their medications are correct and that they're getting the proper support, the physiotherapy, and that the nurses and the ward managers know that there's a link to the PD nurse for that specific patient, just in case they have any queries regarding Parkinson's and how the disease process is affecting their Parkinson's.” (PN4)*  *“I would see the patients are mentioning side effects of the medication […] they have to actually discuss with doctors. So, I have to navigate them back to the doctor, or we do discuss it, but some of the things the doctor has to decide it*.” *(PN2)* |
| …patient care needs (n=4). |
| *“I chose the home safety and care needs monitoring. Because actually our Parkinson's patient, they have, for example, a very high risk of falling, and if we can adapt and teach them and the family how to arrange their houses, it's something that we can proactively do and try to prevent falls, for example, and other stuff.” (PN1)*  *“I'm doing home visits also. So, I go there and sit with them and also their family, and see ‘what can we do’? What can we actually, like, try to adapt to be easier for them and also for their caregivers. Because sometimes that's just a little bit of a change and it's something that can make them comfortable. […] In here we try to do the same, like before they go to their homes, we try to see and to speak and we have a meeting trying to see what can we, they do, […]to proactively try to prevent something.” (PN2)*  *“[…] monitoring home safety and care needs […] because, for my part, it’s often documentation and care needs. Yeah. We're discussing with relatives and with patient.” (PN5)*  *“I put the visiting the patient at home. I feel like in iCare, and besides iCare, I also go to good people's homes and sometimes they feel more comfortable and sometimes they speak some things to me that they don't speak with the doctor at the hospitals, because they're so stressed out because they have to go to the hospital. When they're at home, they feel like they're on their environment. So, it's easier sometimes for them to speak out and sometimes even getting the information. […] Sometimes we have other people there, their spouses, their children, and everyone helps out.” (PN3)* |
| …parameters in a structured manner (n=2). |
| *“You need to be a bit structured for me. You cannot just ask the patient, "how are you?" or, "how has this week gone?" because they don't remember. They cannot always describe really what are their symptoms or what are they leading. So actually, having a routine questionnaire session can be useful also because you can appreciate the differences and you can discuss with the patient about that of course. You can say, "okay. Do you remember two months ago, you told me about this questionnaire, these things?" and you can discuss that. So actually, this assessment or health data can be really important. But you need to have time to do that.” (PN6)*  *“Doing the assessment and their questionnaires before. It's important also to first get some information from them.” (PN3)* |
| Role Cluster #4: Parkinson Nurses as health care coordinators… |
| …of patient pathways (n=6). |
| *“I chose overseeing/scheduling appointments with specialists. From our side, from my experience working with people with Parkinson's and working as a Parkinson's nurse, there's just a lot of things that we would do for our patients. It would be a proactive activity because we would constantly have to check in with them to make sure that they've gotten their schedules either to get their botox or to see other specialists regarding different aspects of their care. […] We are as well monitoring how they get along with their care needs and different aspects of their care that we can't directly provide in our neurology department.” (PN4)*  *“We refer a lot of these patients to public resources or public based care resources. So, a lot of these resources as well might be overwhelmed with the amount of referrals they get. So there has to be kind of a proactivity from our part just to make sure that they are actually going to these schedules and these schedules are not being deferred, because sometimes we would refer them to maybe a urologist, but in the end, they might not ever be seen.” (PN3)*  *“What I feel or how I understand a Parkinson's nurse specialist here is they're the link between the acute hospital and the community. So, since there's such rare posts, there's limited bodies in these posts, we don't have the resources to actually go out and see the patients in their homes. I think that is one of the biggest differences here. What we do here is that we try to communicate the patient's needs to the services that's within their community. So, we would reach out to a public health nurse, alert the public health nurse that a, b, and c needs to be done for this patient, or the community physiotherapist within that area.” (PN5)*  *“I chose hospital discharge guidance. The main reason why I chose that is because, as I said, the Parkinson's nurse specialist here, what they do is that they liaise between the acute hospitals and the community services. So, during discharge, it's important that the Parkinson's nurse are able to guide the patients to say, usually when they're in the hospital, a lot of things change with their Parkinson's. Either they deteriorate or they end up requiring more service, or there's change in their medication.” (PN2)*  *“I think one of the most important parts for us is making sure that when they do get discharged from the hospital, that they have the access to the right people in the community and that they have enough information to manage themselves out in the community.”*  *“(Being) in the community, we're actually doing it here, sometimes they actually go outside too. They go to the home visits. For example, I have a person outside that is [..] having trouble with speaking and everything. So, I called our speech pathologist and they're going there also.” (PN6)*  *“What we try to do is we try to communicate to their GPs so that the person that they see out in the community and use the GP as a resource in that to identify community resources that are available to them. But then also that might be a problem, especially in areas where the resources there are not well funded or are overloaded. So, patients have to wait for x amount of time to be seen before, so that's why they might opt to do a private service or a public service. […] What’s difficult here is trying to get ahold of GPs, like talk to them over the phone, just because that service is quite overloaded. Every communication either has to be through email or a letter, but physically having to be able to talk to their GPs is quite difficult.” (PN1)* |
| …within the multidisciplinary team (n=3). |
| *“I choose overseeing schedule appointment with specialists […] because we are referring the patients’ a lot […] to psychologists, to physio, or different kind of stuff. […]- they really appreciate when we offer them the specialists, because they have the assuredness that they have been taken care of.” (PN2)*  *“Of the kind of main tasks for them is that when a patient gets admitted in the hospital, so what our PD nurse specialist, what they would do is they would go to the wards where they're being treated and make sure that their medications are correct and that they're getting the proper support, the physiotherapy, and that the nurses and the ward managers know that there's a link to the PD nurse for that specific patient, just in case they have any queries regarding Parkinson's and how the disease process is affecting their Parkinson's.” (PN3)*  *“I choose providing a structured care plan, liaison with the care team, and ensuring that patients' needs are addressed. Of course, the care team for me is absolutely important because we know that the patient’s needs [… ] and if we want to ensure that their needs are addressed, we need to have a super strong network. […] And on top level, providing a structured care plan, which can be discussed in a multidisciplinary team […] could be very helpful for the patient because he can feel that he is part of the care and he is not just receiving treatments and so on without knowing what are his or her perspective is”. (PN4)* |
